# Spontaneous symmetry breaking for extreme vorticity and strain in the three-dimensional Navier–Stokes equations

**DOI:** 10.1098/rsta.2021.0051

**Published:** 2022-06-27

**Authors:** Timo Schorlepp, Tobias Grafke, Sandra May, Rainer Grauer

**Affiliations:** ^1^ Institute for Theoretical Physics I, Ruhr-University Bochum, Universitätsstrasse 150, Bochum 44801, Germany; ^2^ Mathematics Institute, University of Warwick, Coventry CV4 7AL, UK; ^3^ Department of Mathematics, TU Dortmund University, Vogelpothsweg 87, Dortmund 44227, Germany

**Keywords:** large deviation theory, instantons, extreme events, optimal control, Navier–Stokes turbulence, vortex sheets

## Abstract

We investigate the spatio-temporal structure of the most likely configurations realizing extremely high vorticity or strain in the stochastically forced three-dimensional incompressible Navier–Stokes equations. Most likely configurations are computed by numerically finding the highest probability velocity field realizing an extreme constraint as solution of a large optimization problem. High-vorticity configurations are identified as pinched vortex filaments with swirl, while high-strain configurations correspond to counter-rotating vortex rings. We additionally observe that the most likely configurations for vorticity and strain spontaneously break their rotational symmetry for extremely high observable values. Instanton calculus and large deviation theory allow us to show that these maximum likelihood realizations determine the tail probabilities of the observed quantities. In particular, we are able to demonstrate that artificially enforcing rotational symmetry for large strain configurations leads to a severe underestimate of their probability, as it is dominated in likelihood by an exponentially more likely symmetry-broken vortex-sheet configuration.

This article is part of the theme issue ‘Mathematical problems in physical fluid dynamics (part 2)’.

## 1. Introduction and motivation

Turbulence is characterized by its tendency to intermittently dissipate energy in very localized and intense events. These extreme events dominate the statistics of quantities such as high order structure functions, and are ultimately responsible for the anomalous scaling of fully developed turbulent flows. It is generally believed that short bursts of intense vortex stretching are the mechanism for the formation of these events.

Taking this as starting point, in this paper, we address the question: what structures are naturally generated in the three-dimensional incompressible Navier–Stokes equations (NSE) to realize events of extreme vortex stretching, strain production and energy dissipation? For this, we are concentrating on small-scale structures that lead to extreme values of the fluid vorticity or its strain. Concretely, we set out to compute the most likely configuration (for a given large-scale stochastic forcing) that realizes a large vorticity or strain value at a single point within the domain, at an instantaneous moment in time, and how the velocity field configuration around this point facilitates the extreme burst.

This question has been discussed in the literature, starting with Novikov *et al.* [1,2], and more recent works that explored extreme vorticity and strain events in very large turbulent simulations [3,4]. These attempts, which solely rely on brute-force direct numerical simulations (DNS), have the intrinsic complication that any extreme realization of an observable will necessarily be very rare, and thus hard to observe. Therefore, exploring extreme events not only requires high numerical resolution but further extremely large datasets, most of which are wasted because they do not exhibit the desired event. On this basis, we instead employ specific rare event techniques [5], in particular stochastic field theory and instanton calculus [6], or equivalently, sample path large deviation theory [7]. The two are intimately connected [6,8], and have proven successful in related fields, such as extreme shocks in Burgers turbulence [9–11], extreme surface heights in the Kardar–Parisi–Zhang (KPZ) equation [12], in ocean waves and tsunamis [13,14], or extreme mechanical forces in grid-generated turbulence [15]. The key idea is to replace the inefficient naive sampling approach by a deterministic optimization problem that yields the maximum-likelihood trajectory of the system that leads to a prescribed rare outcome. The advantage of this method is the fact that it yields the best estimate of the *typical* extreme event in the limit of it becoming increasingly rare, which is the limit we are most interested in, and at the same time also the regime that is hardest to reach via DNS.

As we will discuss later, instanton techniques not only allow for the computation of the limiting most likely path to obtain an extreme event, but further yield estimates for the exponential tail scaling of the observable’s probability density function (PDF). A concrete prediction of our results is the fact that intuitive rotationally symmetric realizations of extreme vorticity outcomes (namely, vortex tubes/filaments) or extreme strain outcomes (namely, colliding or contracting vortex rings) are not necessarily the most likely way to reach extreme values, even if the observable exhibits rotational symmetry. In fact, we present that the rotationally symmetric events become subdominant, particularly for large positive strain values, and are dominated in probability by asymmetric field configurations. In other words, the stochastic instanton undergoes spontaneous symmetry breaking, and the corresponding action exhibits a dynamical phase transition similar to what is observed, e.g. in the KPZ equation [16].

This paper is organized as follows: we discuss the instanton approach, as applied to the NSE, in §2 by first introducing the instanton formalism in §2a in general and subsequently applying it to the NSE in §2b, where we also explain our conditioning on vorticity and strain. The numerical implementation of the corresponding optimization problem is discussed in §2c. In §3a, we show the most likely configuration for extreme vorticity events as obtained by the numerical solution of the instanton problem. Analogous results for extreme strain events are presented in §3b. We will discuss the implication of these results on the likelihood and PDF tail scaling in §3c and then conclude with §4. The electronic supplementary material includes additional, detailed information on the numerical optimization methods that have been used to generate the results of this paper.

## 2. Instantons for the three-dimensional NSE

The three-dimensional incompressible NSE on a domain $\Omega \subset R^{3}$, given by

2.1 $$\begin{array}{rl} & \partial_{t}u+(u\cdot \nabla )u=-\nabla P+\nu \Delta u+\eta ,\\ & \nabla \cdot u=0\\ \mathrm{and}\; & u(\cdot ,-T)=u_{0}, \end{array}\}$$

describe the spatio-temporal evolution of a velocity field $u:\Omega \times [-T,0]\mapsto R^{3}$, where P(x,t) is the pressure field, η(x,t) is the stochastic forcing term, ν>0 is the kinematic viscosity and $u_{0}$ is a deterministic initial condition. We restrict ourselves to a periodic domain $\Omega ={\left[0,l\right]}^{3}$, and consider a white-in-time, spatially stationary and solenoidal Gaussian forcing acting only on large scales as specified by the spatial covariance $\chi :\Omega \to R^{3\times 3}$

2.2 $$\left\langle \eta (x,t)\eta^{⊤}(x^{\prime },t^{\prime })\right\rangle =\chi (x-x^{\prime })\delta (t-t^{\prime }).$$

Additionally requiring the forcing to be statistically isotropic reduces the possible forms of χ to [17]

2.3 $$\chi (x)=f(\|x\|)\text{Id}+\frac{1}{2}\|x\|f^{\prime }(\|x\|)\left[\text{Id}-\frac{xx^{⊤}}{\|x{\|}^{2}}\right],$$

where $\text{Id}\in R^{3\times 3}$ denotes the identity matrix on $R^{3}$, and f:[0,∞)→R is an arbitrary function, which we choose as

2.4 $$f(r)=\chi_{0}\exp\left\{-\frac{r^{2}}{2\lambda^{2}}\right\}$$

for simplicity, with a correlation length λ of the order of the domain size l.

Extreme events in the NSE have been explored extensively in the literature. Particularly worth mentioning in connection with the instanton calculus is the work of Novikov *et al.* [1,2]. They considered the conditionally averaged vorticity field, i.e. the average realization of the vorticity field conditioned on a specific outcome of vorticity ω(x,t=0) at a given point x. These fields, parametrized by ω, were obtained by performing many DNS, and averaging conditioned on the intended outcome. This procedure is closely related to the filtering approach [18] discussed in §3a and demonstrates the relevance of instanton solutions in real flows.

The structure of instanton solutions is of particular importance. As an example serves the observation that the rotational symmetric vorticity instanton in the two-dimensional NSE has no relevance at all [19]. Only taking into account symmetry-breaking angle-dependent contributions results in an effective action suitable for the instanton calculus.

**Figure 1.**
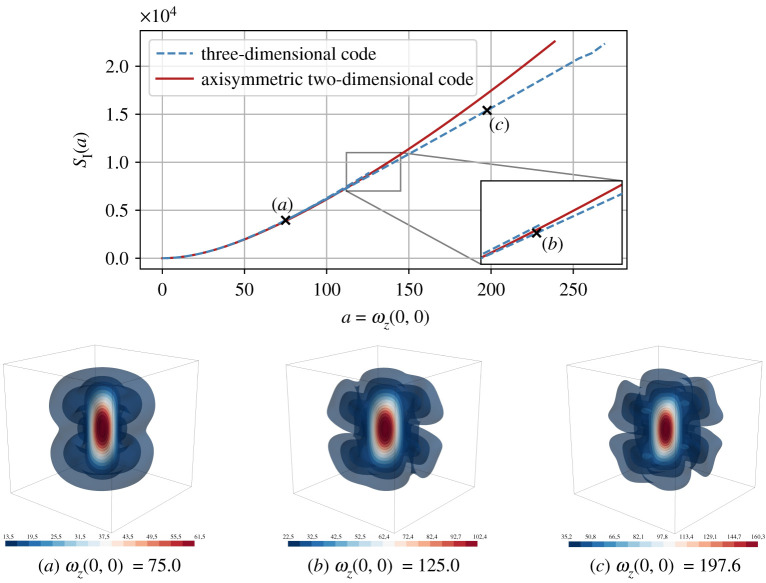
(*a*–*c*) Results of the full three-dimensional and axisymmetric instanton computations for the vorticity observable $\omega_{z}(0,0)$. The plot in the top row shows the action $S_{\text{I}}(a)$ at all critical points of the action that were found in our numerical experiments for different values of the final-time constraint $\omega_{z}(0,0)=a$. The bottom row shows isosurfaces of the vorticity of the final-time configuration of the obtained instanton fields for different observable values as indicated in the top plot. Qualitatively, the field configurations that we observe are vortex tubes in all cases. However, the three-dimensional computations show that a second branch that breaks full rotational symmetry and reduces to reflection symmetry dominates the fully symmetric branch in probability and splits off at $a_{c}\approx 85$. (Online version in colour.)

**Figure 2.**
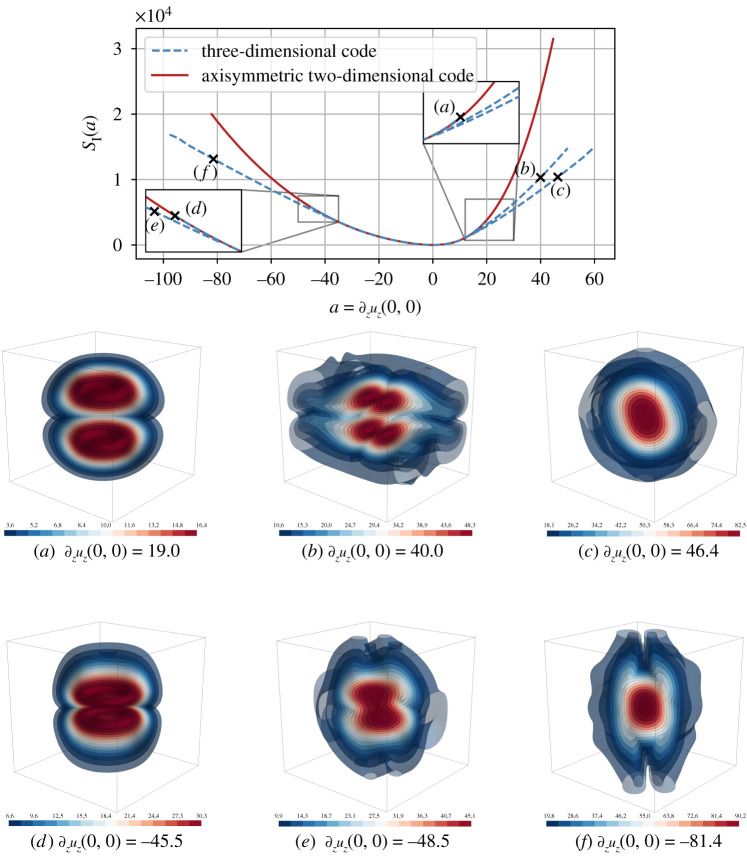
Results of the axisymmetric and full three-dimensional instanton computations for the strain observable $\partial_{z}u_{z}(0,0)$. As in figure 1, the top plot shows the action at all critical points that were found numerically for different observable values, and the two bottom rows show isosurfaces of the vorticity of the final-time configuration of the indicated instanton fields. Note that, contrary to the vorticity instanton, we find a qualitative difference between the rotationally symmetric strain instanton consisting of two counter-rotating vortex rings (*a* and *d*) and a dominant, symmetry-breaking instanton branch that consists of thin vortex sheets (*c*, *e* and *f*). Furthermore, for large positive strain, we find a third, subdominant branch with a quadrupole-like symmetry (*b*). (Online version in colour.)

In the case of the KPZ equation, symmetry breaking (or dynamical phase transition) has been demonstrated as the mechanism to generate the relevant instanton for obtaining the correct tail asymptotics [16]. Here, we make similar observations: symmetry breaking is essential to compute the relevant instanton with a pancake- or sheet-like structure (figures 1 and 2). Whether these structures are related to the recently discovered confined vortex surfaces [20] and the tangential discontinuity of vortex sheets [21] poses a challenging question.

### (a) Stochastic action and minimizers

In this section, we briefly and formally introduce the instanton formalism for stochastic partial differential equations (SPDEs) and comment on the applicability of the method in the context of Navier–Stokes turbulence to compute maximum-likelihood space–time realizations of extreme events. For a generic SPDE for $u:\Omega \times [-T,0]\to R^{3}$

2.5 $$\begin{array}{r} \begin{array}{rl} & \partial_{t}u(x,t)+N(u(\cdot ,t))(x)=\sqrt{\varepsilon }\eta (x,t)\\ \mathrm{and}\; & u(\cdot ,-T)=u_{0}, \end{array}\} \end{array}$$

with a Gaussian forcing correlated according to (2.2) and noise strength ε>0, expectations of a functional F with respect to the process u can formally be computed as a path integral

2.6 $$\begin{array}{rl} \left\langle F[u]\right\rangle & \;=\int D\eta \;F[u[\eta ]]\;e^{-(1/2\varepsilon )\int_{-T}^{0}{\left(\eta ,\chi^{-1}*\eta \right)}_{L^{2}(\Omega ,R^{3})}\;dt}\\ & \;=\int_{u(\cdot ,-T)=u_{0}}Du\;F[u]J[u]\;e^{-(1/\varepsilon )S[u]}, \end{array}$$

where ∗ denotes spatial convolution and $\chi^{-1}$ is the convolutional inverse of the forcing correlation function χ. The Jacobian J[u] is given by $J[u]=\exp\{\frac{1}{2}\int_{-T}^{0}\text{tr}\;\nabla N(u)\;\text{d}t\}$ and S is the classical Onsager–Machlup [22] or Freidlin–Wentzell [7] action functional

2.7 $$S[u]=\int_{-T}^{0}L(u,\partial_{t}u)\;dt=\frac{1}{2}\int_{-T}^{0}{\left(\partial_{t}u+N(u),\chi^{-1}*[\partial_{t}u+N(u)]\right)}_{L^{2}(\Omega ,R^{3})}\;dt,$$

of the process u. In the case of a degenerate forcing, as in our specific application, we set S[u]=+∞ if the trajectory does not lie in the image of the spatial convolution with χ. Suppose now that we are interested in evaluating the probability of measuring particular values of an *observable* O of the final time configuration u(⋅,t=0) in a subset A⊂R. Then, in the small noise limit ε→0, the conditional path density and the probability will be dominated by the least unlikely path, in the sense that

2.8 $$\begin{array}{r} P(O[u(\cdot ,0)]\in A)=\left\langle 1_{\left\{O[u(\cdot ,0)]\in A\right\}}\right\rangle \overset{\varepsilon \to 0}{≍}\exp\{-\frac{1}{\varepsilon }\underset{\begin{array}{c} \tilde{u}(\cdot ,-T)=u_{0}\\ O[\tilde{u}(\cdot ,0)]\in A \end{array}}{\inf}S[\tilde{u}]\}, \end{array}$$

where $1_{\left\{\cdot \right\}}$ denotes the indicator function and ‘≍’ stands for log-asymptotic equivalence (i.e. the logarithms of both sides are equal up to first order [23]). This follows formally by applying Laplace’s method to the path integral (2.6), or more rigorously by Freidlin–Wentzell theory [7]. We denote by $u_{\text{I}}$ the field configuration for which the functional S attains its global minimum for the given boundary conditions, i.e. $u_{\text{I}}$ solves the following minimization problem:

2.9 $$\left\{ \begin{array}{ll} \min_{u} & S[u],\\ \text{subject to } & u(\cdot ,-T)=u_{0},\\ & O[u(\cdot ,0)]\in A. \end{array}\right.$$

We call $u_{\text{I}}$ the *instanton* and $S_{\text{I}}=S[u_{\text{I}}]$ the instanton action, and can thus gain access to limiting estimates of probabilities or PDFs in the small noise limit ε→0 by solving the *deterministic* optimization problem of finding $u_{\text{I}}$ via (2.9). For the estimation of PDFs $\rho_{O}(a)$, the target set is A=[a,a+da] and hence the optimal field configuration is sought by minimizing the action functional S[u] subject to the constraint O[u(⋅,0)]=a, which is equivalent to maximizing its probability in path space. Introducing $p=\chi^{-1}*[\partial_{t}u+N(u)]$, we can reformulate (2.9) as a the minimization problem with respect to p given by

2.10 $$\left\{ \begin{array}{ll} \min_{p} & S[p]=\min_{p}\frac{1}{2}\int_{-T}^{0}{\left(p,\chi *p\right)}_{L^{2}(\Omega ,R^{3})}\;dt,\\ \text{subject to } & \partial_{t}u+N(u)=\chi *p,\\ & u(\cdot ,-T)=u_{0},\\ & O[u(\cdot ,0)]=a, \end{array}\right.$$

with u=u[p] being a function of the control p that is given by solving the PDE $\partial_{t}u+N(u)=\chi *p$, $u(\cdot ,-T)=u_{0}$, forward in time.

We denote by $p_{\text{I}}$ the optimal control and by $u_{\text{I}}=u[p_{\text{I}}]$ the associated optimal state. Then, the necessary optimality conditions for (2.10) (derived by using a formal Lagrange approach and eliminating the adjoint state variable afterwards, compare §3a in the electronic supplementary material) yield the instanton equations

2.11 $$\begin{array}{rl} & \partial_{t}u_{\text{I}}+N(u_{\text{I}})=\chi *p_{\text{I}},\\ & \partial_{t}p_{\text{I}}-{\left(\nabla N(u_{\text{I}})\right)}^{⊤}p_{\text{I}}=0,\\ & u_{\text{I}}(\cdot ,-T)=u_{0},\;O[u_{\text{I}}(\cdot ,0)]=a\\ \mathrm{and}\; & p_{\text{I}}(\cdot ,0)=-{{\frac{\delta O}{\delta u}}^{⊤}|}_{u_{\text{I}}(0)}F_{\text{I}}. \end{array}\}$$

Here, $F_{\text{I}}$ is a Lagrange multiplier to enforce the final time constraint.

Note that we started our considerations by expressing the probability of an event via a path integral. The final object we obtain though, namely the instanton, is interesting in its own right in that it is exactly the *most likely* realization of the outcome we set out to observe, regardless of whether it indeed represents the *typical* realization of that outcome. The crucial subtlety here is that for a common event there are usually a multitude of possible histories for its creation, while an extreme outlier event is usually driven by a very specific and reproducible chain of events. The *average* field configuration realizing a moderate vorticity, say, will in general be very different from its *most likely* configuration, and in fact is rather meaningless, as it averages over many different and unrelated physical mechanisms. For *extreme* events, on the other hand, the two notions coincide, and the most likely conditioned configuration precisely corresponds to the conditioned field average.

The connection to Freidlin–Wentzell theory [7] and large deviation theory rare events algorithms [8] allows us to make this notion rather precise: the large deviation limit in the set-up that was outlined above is correct in the small noise limit. Through a suitable rescaling of (2.1), this limit is, in the first instance, equivalent to the low Reynolds number limit for the NSE: non-dimensionalizing all variables via $\tilde{x}=x/x_{0}$, $\tilde{t}=t/t_{0}$, $\tilde{u}=ut_{0}/x_{0}$, $\tilde{P}=Pt_{0}^{2}/x_{0}^{2}$ and $\tilde{\eta }=\eta t_{0}^{1/2}/\chi_{0}^{1/2}$ and choosing $t_{0}=x_{0}^{2}/\nu$ yields

2.12 $$\begin{array}{rl} & \partial_{t}u+(u\cdot \nabla )u=-\nabla P+\Delta u+\sqrt{\varepsilon }\eta ,\\ & \nabla \cdot u=0\\ \mathrm{and}\; & u(\cdot ,-T)=u_{0}, \end{array}\}$$

in the new variables. Here, $\varepsilon =\chi_{0}x_{0}^{4}\nu^{-3}=Re^{3}$ if $x_{0}$ is taken to be the characteristic length scale of the forcing and the characteristic velocity $u_{0}$ for the Reynolds number $Re=u_{0}x_{0}/\nu$ is chosen as $u_{0}={\left(\chi_{0}x_{0}\right)}^{1/3}$. This shows that as Re→0, the instanton prediction for quantities such as $\rho_{O}(a)$ will become asymptotically exact for the full range of the PDF. In contrast to this set-up, we are interested in flows at a given and possibly large Reynolds number. This can be achieved by realizing that the small noise (small Re) limit can be exchanged for an extreme event limit (see remark 1 in [24]): if the length and time scales are chosen such that $\varepsilon =\chi_{0}/(\nu a_{0}^{2})$, and we focus on an event with $|O[u(\cdot ,0)]|=a_{0}\gg \sqrt{\chi_{0}/\nu }$ (for an observable with dimension velocity over length), for a given Reynolds number, the instanton estimate for the typical event itself and its probability will be accurate for sufficiently large $a_{0}$ or sufficiently extreme events. For high Re, these observables must take very extreme values for the scaling limit to apply, making it very hard to observe in DNS. As a consequence and as we will confirm numerically in §3, the instanton scaling is readily reached for small Reynolds numbers, while it is entirely out of reach of direct sampling for high Re, because we are probing the tail scaling for extremely unlikely events. This associates instantons with structures deep within the dissipation range. We remark that the formation of these nearly singular dissipative structures (see §3a,b) might be the cause of the dissipation anomaly [25].

### (b) Instanton equations for Navier–Stokes with axisymmetric observables

For the NSE (2.12), the instanton equations (2.11) can be written as

2.13 $$\begin{array}{rl} & \partial_{t}u_{\text{I}}+P[(u_{\text{I}}\cdot \nabla )u_{\text{I}}]-\Delta u_{\text{I}}=\chi *p_{\text{I}},\\ & \partial_{t}p_{\text{I}}+P[(u_{\text{I}}\cdot \nabla )p_{\text{I}}+{\left(\nabla p_{\text{I}}\right)}^{⊤}u_{\text{I}}]+\Delta p_{\text{I}}=0,\\ & u_{\text{I}}(\cdot ,-T)=u_{0},\;O[u_{\text{I}}(\cdot ,0)]=a\\ \mathrm{and}\; & p_{\text{I}}(\cdot ,0)=-P\left[{{\frac{\delta O}{\delta u}}^{⊤}|}_{u_{\text{I}}(0)}F_{\text{I}}\right] \end{array}\}$$

in coordinate-free form with $\nabla \cdot u_{\text{I}}=\nabla \cdot p_{\text{I}}=0$. Here, the Leray projection $P=\text{Id}-\nabla \Delta^{-1}\nabla \cdot$ onto the divergence-free part of a vector field [26] has been introduced in order to eliminate the pressure from the equations of motion and conveniently handle the incompressibility constraint within the general framework that has been presented in the previous section. A detailed derivation of (2.13) is carried out in §4a of the electronic supplementary material.

We note that it is very challenging to make mathematically rigorous statements about the (unique) solvability of the corresponding minimization problem (2.10) for the NSE, as well as about the question of local versus global solutions. We do not attempt to do this here. Instead, for assessing the validity of our results, we rely on the following observations: (i) for the simplified case of the heat equation, the equation for p in (2.13) is independent of u and the instanton equations can be solved directly without an iterative procedure and thus that problem has a unique global minimizer; (ii) for the NSE, we can get a good indication whether a numerically found solution to the minimization problem is indeed globally optimal by comparing with the PDF as obtained via DNS; (iii) for the NSE, we numerically validated our claim concerning the global optimality by restarting our optimization algorithms at various points. Within this reasoning, we will assume in the numerical results that we have found globally optimal solutions.

We are interested in extreme events for two distinct one-dimensional observables: the vorticity $O_{1}[u(\cdot ,0)]={\left(\nabla \times u\right)}_{z}(0,0)=\omega_{z}(0,0)$, and the strain $O_{2}[u(\cdot ,0)]=\partial_{z}u_{z}(0,0)$. These observables correspond to the transversal and longitudinal components, respectively, of the velocity gradient tensor. Due to statistical isotropy and spatial stationarity, we are free to choose the respective z components as observables, as well as the origin x=0 as the arbitrary point where the observables are evaluated.

Both observables naturally define a distinguished axis, around which the problem is rotationally symmetric. In particular, not only are the NSE rotationally symmetric but also their corresponding action *including the conditioning on the observable* is invariant under rotation around this axis. It is therefore intuitive to search for a rotationally symmetric minimizer, and this is indeed also the nature of the structures that immediately come to mind for vorticity and strain: strong vorticity will be observed at the core of a particularly strong vortex filament, while large strain occurs at points such as the centre of the collision of two vortex rings. Of course, it is not necessarily true that a rotationally symmetric optimization problem has a rotationally symmetric minimizer.

Because of this fact, we set out to search for multiple, possibly distinct minimizers of the action: one for which we artificially enforce rotational symmetry, and potentially others for which no symmetry is enforced. The former case reduces the problem to (2+1) dimensions in (r,z,t) for cylindrical coordinates (r,θ,z) in space. In this coordinate system, and using the vorticity-streamfunction formulation for axisymmetric flows [27], the *axisymmetric* instanton equations are

2.14 $$\begin{array}{rl} & D_{t}u_{\theta }+\frac{1}{r}u_{r}u_{\theta }-Lu_{\theta }={\left[\chi *p\right]}_{\theta },\\ & D_{t}\omega_{\theta }-\frac{1}{r}u_{r}\omega_{\theta }-\frac{1}{r}\partial_{z}(u_{\theta }^{2})-L\omega_{\theta }={\left[(\nabla \times \chi )*p\right]}_{\theta },\\ & D_{t}p_{\theta }+\frac{1}{r}(2u_{\theta }p_{r}-u_{r}p_{\theta })+Lp_{\theta }=0\\ \mathrm{and}\; & D_{t}\sigma_{\theta }-\frac{1}{r}\partial_{z}(u_{\theta }p_{\theta })+\partial_{z}u_{\theta }\partial_{r}p_{\theta }-\partial_{r}u_{\theta }\partial_{z}p_{\theta }\\ & \;\;+2(\omega_{\theta }+2\partial_{r}u_{z})\partial_{r}p_{r}+\frac{2}{r}\partial_{r}u_{z}p_{r}+\left(2\partial_{z}u_{z}+\frac{u_{r}}{r}\right)(2\partial_{z}p_{r}-\sigma_{\theta })+L\sigma_{\theta }=0, \end{array}\}$$

where $D_{t}=\partial_{t}+u_{r}\partial_{r}+u_{z}\partial_{z}$ is the axisymmetric convective derivative, $L=(1/r)\partial_{r}(r\partial_{r})-(1/r^{2})+\partial_{zz}$ is an elliptic operator stemming from the vector Laplacian in cylindrical coordinates, and σ=∇×p is the vorticity of the adjoint field. In this formulation, the r and z components of the fields are reconstructed by solving $L\psi =-\omega_{\theta }$ for the streamfunction ψ and computing $u_{r}=-\partial_{z}\psi$ and $u_{z}=(1/r)\partial_{r}(r\psi )$.

The derivation of (2.14), as well as the spatio-temporal boundary conditions of the axisymmetric instanton fields, can be found in §5a of the electronic supplementary material.

### (c) Numerical procedure

We consider the problem of minimizing the action functional S[u] given by (2.7) subject to a final time constraint O[u(⋅,0)]=a∈R. Here, we briefly outline the numerical procedure that we use to compute axisymmetric and fully three-dimensional solutions.

We interpret the minimization problem within the framework of PDE-constrained optimal control (e.g. [28,29]): we introduce the control variable p as discussed above and consider the optimization problem (2.10). This is an optimal control problem with distributed control as the control p enters the PDE on the right-hand side as a source term. The velocity field u=u[p] corresponds to the state variable. By treating u as a function of p, we can follow the so-called reduced approach in optimal control theory and view the optimal control problem as a problem of the argument p only. We can recast (2.10) into a sequence of unconstrained optimization problems using the augmented Lagrangian method [30]: For a sequence of positive penalty parameters $\left(\mu^{\left(m\right)}\right)$ with $\mu^{\left(m\right)}\to \infty ,$ we minimize

2.15 $$L_{\text{A}}[p,F,\mu ]=S[p]+F(O[u[p](\cdot ,0)]-a)+\frac{\mu }{2}{\left(O[u[p](\cdot ,0)]-a\right)}^{2},$$

while updating the Lagrange multiplier F via $F^{\left(m+1\right)}=F^{\left(m\right)}+\mu^{\left(m\right)}(O[u[p^{\left(m\right)}](\cdot ,0)]-a)$.

In other words, for each penalty parameter $\mu^{\left(m\right)}$, we need to solve a minimization problem, which in turn requires an iterative scheme. The computational costs can be reduced by using warm starts. This procedure allows us to compute instantons for a specified observable value a. This is in contrast to the optimization approach by Chernykh & Stepanov [10] and others. There, the instanton equations, compare (2.11) for a generic SPDE, are solved by an iterative procedure during which the Lagrange multiplier F is kept fixed and the value of a is allowed to change. This again produces a solution to the instanton equations, including a matching pair $\left(F_{\text{I}},a\right)$, but the value of a is not known *a priori*. This practical approach is convenient and computationally cheaper if there is a bijective map between F and a and one is interested in solving the instanton equations over a wide range of values of a. Our approach is more general and to be preferred if (i) there are multiple local minimizers, and the map F↦a becomes multi-valued and (ii) there are observable regions where the action fails to be convex and the F-a-duality breaks down [31].

To minimize (2.15) for a given value $\mu^{\left(m\right)}$, we employ gradient-based methods. As an improvement over a simple gradient descent (which, preconditioned with $\chi^{-1}$, reduces to an iterative, fixed point-like solution of the instanton equations), we use the L-BFGS algorithm (e.g. [32]). This significantly speeds up the computation for the fully three-dimensional instantons. The L-BFGS scheme is a limited-memory variant of one of the most popular quasi-Newton schemes, the BFGS scheme, named after Broyden, Fletcher, Goldfarb and Shanno. Quasi-Newton schemes only require gradient information (in contrast to the second-order derivative information needed for Newton) and typically show super-linear convergence (whereas the gradient scheme only converges linearly with rates that are often very close to 1 for ill-conditioned problems). Appropriate step sizes for the optimization algorithm are determined by an Armijo line search using backtracking [32]. This very popular condition guarantees sufficient decrease that is proportional to the step length. For the evaluation of the gradient, we use an adjoint approach: the gradient is given as $\delta L_{\text{A}}/\delta p=\chi *(p-z)$, where the adjoint state z solves the backward equation $\partial_{t}z-{\left(\nabla N(u[p])\right)}^{⊤}z=0$ with final condition $z(\cdot ,0)=-{\left(\delta O/\delta u{|}_{u[p](\cdot ,0)}\right)}^{⊤}(F+\mu (O[u[p](\cdot ,0)]-a))$. Thus, each gradient evaluation requires to solve a PDE forward in time to determine u[p] and then backwards to compute z. All of this is described in detail in §3 in the electronic supplementary material.

We use two different flow solvers within the described optimization framework: a (2 + 1)-dimensional axisymmetric code as well as a (3 + 1)-dimensional code for the full problem. The (2 + 1)-dimensional code is necessary to compute solutions of the minimization problem under the additional constraint of preserving axisymmetry. For the (3 + 1)-dimensional code, the rotationally symmetric instanton eventually ceases to be a local minimizer of the action as there are unstable directions that break symmetry. Symmetrization stabilizes the configurations and allows us to get access to the associated action. In other words, after symmetry breaking, the axisymmetric configuration ceases to be a minimizer of the full optimization problem, but remains a (local) minimizer of the axisymmetric optimization problem. The axisymmetric code is based on [33]: we use a Leapfrog scheme in time and symmetric second-order finite differences on a regular r-z-grid in space, with a resolution of $n_{t}=1024$ and $n_{r}=n_{z}=256$. The diffusion term is discretized semi-implicitly to avoid a severe CFL constraint. Consequently, in each time step, we need to solve a Helmholtz-like equation to update the fields, for which we use a multi-grid algorithm (e.g. [34]). The polar convolutions with χ are evaluated by means of fast Hankel transforms [35,36].

The full (3+1)-dimensional flow solver uses a pseudo-spectral method in space and the Heun scheme in time, with an integrating factor for the diffusion term. Thus, we again avoid a strict CFL constraint. We run a resolution of $n_{t}=512$ and $n_{x}=n_{y}=n_{z}=128$. For speed up, we implemented this on a GPU using the CUDA API. To fit a full (3+1)-dimensional optimization problem on a single GPU, memory reduction techniques as described in [37] were necessary.

## 3. Results

In the following, we show the outcome of our numerical computations, beginning with the instanton configurations before and after symmetry breaking for both vorticity and strain. We then discuss implications on the tail scaling of the PDFs, in particular for large positive strain.

### (a) Extreme vorticity events

Selecting $\omega_{z}(0,0)=a$ as our observable, we use the above formalism to numerically solve the optimization problem (2.9). The result is the most likely configuration to realize an extreme vorticity outcome at final time. Note that this computation is independent of the choice of the Reynolds number. The Reynolds number, or equivalently ε, only determines whether a chosen observable a is rare, and thus whether the instanton formalism has any relevance for events of this size. As shown in figure 1, the most likely configuration to realize an extreme vorticity corresponds to a vortex filament with an added swirl component. We first show, in the top row of figure 1, how the full three-dimensional and the axisymmetric code find the same minimizer for low values of a, but find different minimizers for high values. Configuration (*a*), at a=75.0, is still in the regime where the global minimizer is rotationally symmetric. At configuration (*b*), for a=125.0, the symmetry-broken branch has already appeared, but is still very close to the symmetric one. Configuration (*c*), at a=197.6, is in a regime where the symmetry-broken minimizer clearly dominates the symmetric minimizer. The asymmetric minimizing configurations correspond to vortex tubes with a symmetry-breaking helical vortex structure around it that displays only reflection symmetry instead of full axial symmetry. Due to the symmetry of the minimization problem under reflection with respect to the z=0 plane, the behaviour is identical for negative a, with a mere sign-flip in ω (not shown).

Note that around the point of symmetry breaking, the full three-dimensional code picks up both the symmetric and asymmetric minimizers until the symmetric configuration eventually becomes unstable, as indicated by the two blue dashed lines in the inset of figure 1 (top), where the upper line corresponds to the rotationally symmetric local minimizer of the full three-dimensional code. There is a small difference between the rotationally symmetric minimizer of the full three-dimensional code, and the same minimizer for the axisymmetric code, which is the result of numerical differences in the integration schemes and coordinate systems.

**Figure 3.**
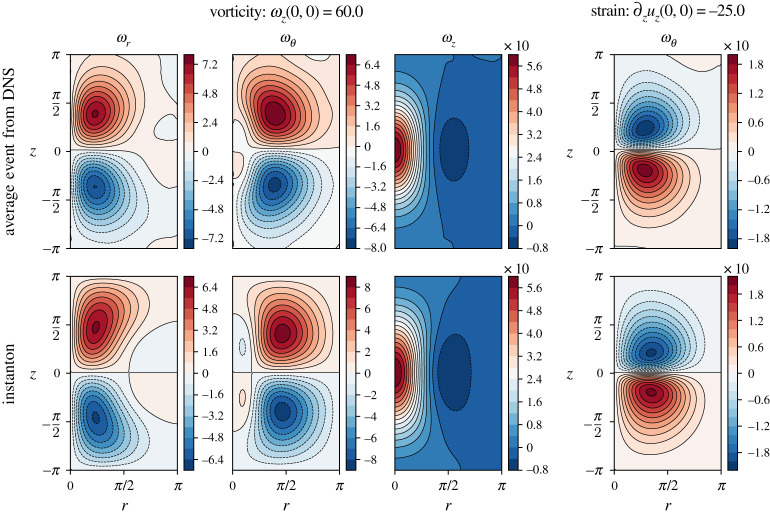
Comparison of the final-time field configuration of *axisymmetric* vorticity and strain instantons (bottom row) to conditional averages of DNS data for the same prescribed observable values at the origin as the instanton fields (top row). The left three columns show all components of the vorticity in cylindrical coordinates for an event with a prescribed value of $\omega_{z}(x=0,t=0)=60.0$ at the origin. The right-most column only shows the θ component of the vorticity of an event with $\partial_{z}u_{z}(x=0,t=0)=-25.0$ since the $\omega_{r}$ and $\omega_{z}$ components are negligibly small. The conditional averages of the DNS data include an angle averaging procedure in θ, and events with suitable observable values at x≠0 were shifted onto the origin. For the displayed vorticity event, approximately $8.4\times 10^{3}$ single events as obtained from DNS of (2.12) with a forcing strength of ε=250 were averaged, whereas the strain event is an average of approximately $5.1\times 10^{3}$ events in the same dataset. (Online version in colour.)

We can compare the instanton configuration against structures observed in DNS, conditioned on observing an extreme vorticity event [1]. The result of this ‘filtering’ procedure [18] is shown in figure 3 (left three columns) for the axisymmetric configuration only. Concretely, this compares an instanton for $\omega_{z}=a=60.0$, which would be located left of configuration (*a*) in figure 1, in cylindrical coordinates, against the conditional average of DNS data at ε=250, conditioned on $\omega_{z}=60.0$. To compute this average, we integrate $10^{4}$ independent realizations of the three-dimensional NSE (2.12) on $\Omega ={\left[0,2\pi \right]}^{3}$ for a total time of T=1 used in all computations throughout this paper (which is much larger than the large eddy turnover time $T_{\text{LET}}\approx 0.1$ for this ε). Exploiting the statistical isotropy and homogeneity of the system in order to increase the sample size, we analyse the final field configuration for events with |‖ω(x)‖−a|/a<0.01, and then rotate and translate the coordinate system so that the event is located at x=(0,0,0) and points in z-direction. We average $8.4\times 10^{3}$ such events, including averaging in θ for each individual event, to obtain the results of figure 3 (top row). The conditional average obtained in this way agrees excellently with the instanton event for the same vorticity, demonstrating that for this Re the most likely and the average configuration realizing $\omega_{z}=60.0$ are identical, and we are indeed in the large deviation limit.

### (b) Extreme strain events

Performing the same procedure for the strain observable, $\partial_{z}u_{z}(0,0)=a$, we obtain a richer set of outcomes. For the strain, positive and negative observables have different phenomenologies caused by the advection term (e.g. [38]), but both eventually undergo symmetry breaking. As visible in figure 2 (top), the earliest and most dramatic symmetry breaking is observed for the positive tail of the strain, where an asymmetric branch splits off already at $a_{c}\approx 14$. Here, the symmetric configuration (*a*), consisting of two counter-rotating, contracting vortex rings, transitions for higher a into an asymmetric sheet-/pancake-like structure (*c*). We additionally observe a further subdominant symmetry-breaking branch of quadrupole-like configurations (*b*). It is of course difficult to exclude the existence of further subdominant local minimizers, but our numerical experiments where we started the optimization algorithm either at a random initial condition for the control or at perturbed solutions of previous problems did not show indications of further branches in the considered observable range.

The negative tail has qualitatively similar behaviour at different values a: the symmetric configuration (*d*), corresponding to two colliding vortex rings with opposite orientation, breaks away at $a_{c}\approx -38$ into more complicated and asymmetric vortex sheet configurations (*e*) and (*f*).

The vortex ring structures that we encountered are ‘trivial’ solutions of the instanton equations (2.14) in cylindrical coordinates in the sense that they satisfy $\omega_{r}=\omega_{z}\equiv 0$, which does not yield the global minimum of the full action at large observable values. Interestingly, field configurations of this type have previously been found as maximizers of the enstrophy growth rate in [39]. On the other hand, sheet-like structures, as in the symmetry-breaking case, have been observed as the most intense dissipative structures already in [40] and in recent spectral simulations using $8192^{3}$ grid points [41].

We further compare the rotationally symmetric strain instanton to the conditional average from DNS in figure 3 (right-most column). Here, with the dataset and procedure as for the vorticity, we only compare the $\omega_{\theta }$-component in cylindrical coordinates, with excellent agreement between the minimizer and the observed conditionally average event realizing the strain value of $a=\partial_{z}u_{z}(0,0)=-25.0$. For the vortex ring configuration, all other components are negligibly small ($\approx 10^{-4}$ for the three-dimensional instanton code due to numerical noise, $\approx 10^{-1}$ due to statistical noise in the DNS average). We do not compare the symmetry-broken instantons with conditional averages, since this would require a much larger dataset, where each event, instead of being averaged over θ, is additionally aligned in angular direction, using e.g. the eigenvectors of the velocity gradient tensor.

### (c) Extreme event probabilities

The derivation of the instanton formalism in §2, and in particular equation (2.8), make obvious that the instanton not only represents the most likely extreme event but further allows us to estimate its probability, which scales exponentially with the instanton action. In this section, we compare this prediction for the exponential scaling of the tail with PDFs obtained from DNS, and in particular demonstrate how the symmetry-broken instanton predicts the correct tail scaling for the PDFs, while the axisymmetric instantons dramatically underestimate the likelihood of large strain events. We concentrate on the positive tail of the strain observable in particular, since there the symmetry is broken the earliest and the difference in slope is the clearest.

**Figure 4.**
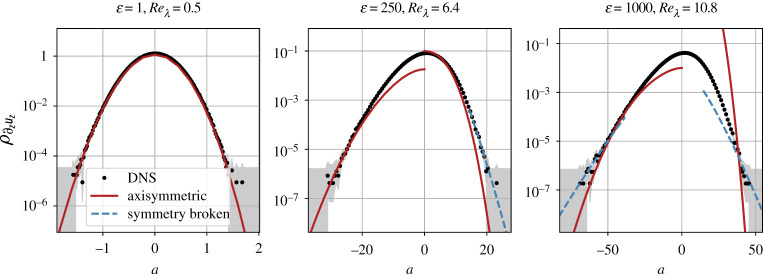
Comparison of the instanton prediction $\propto \exp\{-\varepsilon^{-1}S_{\text{I}}(a)\}$ for the strain PDF $\rho_{\partial_{z}u_{z}}$ to DNS data at different forcing strengths ε or Taylor–Reynolds numbers $Re_{\lambda }$. The dots show the DNS histogram, with a 95% Wilson score interval [42,43] shaded in grey. The solid lines show the PDF prediction as obtained from the axisymmetric instanton configurations, whereas the dashed lines show the PDF prediction based on the *lowest* symmetry-broken branch of the instanton action. Note that we are free to shift all individual branches arbitrarily and independently in the vertical direction in the semi-logarithmic PDF plot since we are only interested in asymptotic scaling estimates. Observe in particular that the axisymmetric strain instanton clearly underestimates the right tail even at the small Reynolds numbers considered here. (Online version in colour.)

Figure 4 shows this comparison for three different values ε∈{1,250,1000}, corresponding to three different Taylor–Reynolds numbers $Re_{\lambda }=\sqrt{15\;Re}\in \{0.5,6.4,10.8\}$ (where Re was determined from the root mean square velocity and integral scale of the data). Note that even the highest Re is still comparably low. This is because, as argued in §2a, for higher Re the instanton is so far in the tail that it cannot be detected in DNS.

For each ε, we performed $10^{4}$ pseudo-spectral simulations of the three-dimensional NSE (2.12) at a spatial resolution of $128^{3}$ starting from $u_{0}=0$ at T=−1 until t=0. The final time configurations were subsampled according to the estimated approximate correlation length $\lambda_{\partial_{z}u_{z}}=0.8$ of the observable, and the shaded area indicates a 95% Wilson score interval [42,43] for the PDF estimate based on the DNS data. For the lowest Re, the data are almost Gaussian, and the instanton and PDF agree everywhere. No symmetry breaking is observed. For the two higher Re, instead, the instanton approach only captures the tail scaling correctly, since common strain events are not dominated by the instanton in this case. In the tails, though, the axisymmetric instanton clearly underestimates the probability, while the symmetry-broken instanton is in good agreement. This is particularly clear in the right tail of the right-most panel of figure 4, where the axisymmetric instanton depicted by the red line is far too steep to agree anywhere with the observed tail scaling. This trend continues in fully developed turbulence at higher Re: the analysis of larger DNS, e.g. in [4], shows that the strain PDF tails can in fact be described by stretched exponentials $\propto \exp\{-c_{\pm }|a{|}^{\vartheta_{\pm }}\}$ with exponents $\vartheta_{\pm }<1$, whereas we find that the exponents in both tails derived from the vortex-ring instanton increase monotonically with |a| and saturate above $\vartheta_{+}=2.5$ in the right tail and above $\vartheta_{-}=2$ in the left tail. By contrast, while the $S_{\text{I}}(a)$-curve that we obtained for the symmetry-broken instantons is still convex in the observable range that we were able to consider at the given resolution, the exponents $\vartheta_{\pm }$ are monotonically decreasing in |a| for this branch and decay below 1.5 for both positive and negative strain.

For the vorticity observable of the same DNS dataset at ε∈{1,250,1000}, we observe the same qualitative results (not shown): at the lowest Re, the instanton again perfectly describes the PDF, whereas the range of validity of the estimate transitions into the tails at higher Re. Here, however, because the symmetry breaking occurs at relatively higher a and leads to a less dramatic difference in scaling, it is hard to draw as clear a conclusion as in the strain case.

## 4. Conclusion

In this paper, we set out to numerically compute maximum-likelihood realizations of extreme vorticity and strain events in the stochastic incompressible three-dimensional NSE. As an alternative and complement to direct sampling approaches, we rephrased the problem into a deterministic variational framework using sample path large deviation theory, which is particularly suited for rare and extreme events. This led us to consider a (3 + 1)-dimensional optimization problem with final-time constraints to enforce large observable values, which we were able to solve using tools from PDE-constrained optimization. For both observables considered here, we observe symmetry breaking of the minimizers: the vortex filaments that lead to large values of the vorticity reduce from axial to reflection symmetry, and the vortex rings that realize large strain transition to a pancake-like vortex sheet structure. For positive strain in particular, we demonstrated that the symmetry-broken minimizer clearly dominates the symmetric one and can in fact be confirmed to yield the correct scaling of DNS PDFs at suitable Re, in contrast to the axisymmetric one.

The possibility to access the most extreme events in Navier–Stokes turbulence without sampling is attractive. Despite the fact that the optimization problem (2.9) to be solved is massive, with fields of size $512\times 128^{3}$, and a single iteration of the minimization algorithm corresponding to a forward integration of the NSE, and an equally sized backward propagation, we show that this effort pays off for extreme outlier events: obtaining these same configurations traditionally necessitates either millions of samples of the stochastic NSE (for lower Re), or the regime is completely inaccessible as the events are entirely too rare and extreme to be observed (for higher Re). While one could try to formulate reduced problems in effective coordinates, for example as in [44], our approach yields the most likely configuration without any *a priori* assumptions about its form or physical mechanisms.

In this paper, we only considered the exponential contribution of the minimizer for the PDF. Improved estimates are possible in principle when taking into account the fluctuations around the instantons, as discussed e.g. in [24]. The computational cost of computing this fluctuation determinant is vastly bigger than the already large problem sizes encountered in the optimization problem in this work. For this approach, it is further necessary to integrate out the zero mode associated with the symmetry breaking of the instanton. This correction to the PDF was ignored in this paper.

It would be interesting to determine whether the viscid instanton we discussed here has relevance to inertial range properties of turbulent flow. One possible connection is given by the scaling of velocity gradient moments, which, even in the low Reynolds numbers regime, link dissipative statistics to inertial range properties via so-called fusion rules [45–47]. This possible route towards understanding intermittency is the focus of our future work.

## Data Availability

All necessary data and scripts to generate the figures of this paper are available in the electronic supplementary material [48].
